# Genetic interactions between INPP4B and RAD50 is prognostic of breast cancer survival

**DOI:** 10.1042/BSR20192546

**Published:** 2020-01-10

**Authors:** Xiao Chen, Rutaganda Theobard, Jianying Zhang, Xiaofeng Dai

**Affiliations:** 1 School of Biotechnology, Jiangnan University, Wuxi, China; 2 Henan Institute of Medical and Pharmaceutical Sciences and Henan Key Laboratory of Tumor Epidemiology, Zhengzhou University, Zhengzhou 450001, China; 3 Hospital of Xi’an Jiaotong University, Xi’an, 710061, China; 4 Wuxi School of Medicine, Jiangnan University, Wuxi, China

**Keywords:** Breast cancer, INPP4B, RAD50, SNP

## Abstract

*RAD50* is commonly depleted in basal-like breast cancer with concomitant absence of *INPP4B* and several tumor suppressors such as *BRCA1* and *TP53*. Our previous study revealed that *INPP4B* and *RAD50* interact and such an interaction is associated with breast cancer survival at the transcriptional, translational and genomic levels. In the present study, we explored single nucleotide polymorphisms (SNPs) of these two genes that have synergistic effects on breast cancer survival to decipher mechanisms driving their interactions at the genetic level. The Cox’s proportional hazards model was used to test whether SNPs of these two genes are interactively associated with breast cancer survival, following expression quantitative trait loci (eQTL) analysis and functional investigations. Our study revealed two disease-associating blocks, each encompassing five and two non-linkage disequilibrium linked SNPs of *INPP4B* and *RAD50*, respectively. Concomitant presence of any rare homozygote from each disease-associating block is synergistically prognostic of poor breast cancer survival. Such synergy is mediated via bypassing pathways controlling cell proliferation and DNA damage repair, which are represented by INPP4B and RAD50. Our study provided genetic evidence of interactions between *INPP4B* and *RAD50*, and deepened our understandings on the orchestrated genetic machinery governing tumor progression.

## Introduction

As the second leading cause of deaths worldwide, great attention has been paid in order to reveal the underlying factors that drive the genesis of cancer [1]. Evidences showed that various factors are linked with growth and development of different types of cancer which include mutation [2], radiation [3], inflammatory bowel disease [4], viral [5,6] and bacterial infection [4,7]. Breast cancer is the leading cause of deaths among women with the annual mortality rate being estimated over 570000 worldwide [8,9]. Most breast cancers are sporadic cancers caused by accumulation of acquired yet uncorrected genetic alterations in somatic genes, while other cases are associated with inherited genetic changes in disease predisposing genes [10]. As one of the most common types of genetic variations in human genome, single nucleotide polymorphisms (SNPs) in genes involved in DNA damage repair, metabolism, carcinogen metabolism, cell-cycle control, apoptosis and immunity are likely to be associated with genetic susceptibility to various cancer types including breast cancer [11,12]. So far, common SNPs can account for 18% of breast cancer familial risk among women [13].

SNPs can be located in either coding or non-coding regions. SNPs located in the coding regions are assumed to be able to affect protein production and functionalities, and thus more likely to cause phenotypic changes [14]. SNPs located in the non-coding region are less toxic and more easily to be inherited. However, recent advances suggest that SNPs in the non-coding regions may also play a functional role including, e.g. RNA splicing, genome imprinting, long non-coding RNAs binding etc [11].

*RAD50* is crucial to maintain genomic integrity and prevent tumorigenesis [15]. It is a key protein involved in DNA double-strand breaks repair and frequently deleted in basal-like breast tumors [16]. *RAD50* is a breast cancer susceptibility gene associated with genomic instability [17]. Loss of *RAD50* often co-occurs with deletion of one or more tumor suppressor genes *BRCA1, TP53, PTEN, RB1* and *INPP4B* [18]. *INPP4B* is involved in the control of cell proliferation, cell metabolism and apoptosis [19]. *INPP4B* resides in the PI3K/Pten/mTOR pathway which is a complex network that controls cell proliferation and survival and is deregulated in over 70% of breast cancers [20]. Moreover, *INPP4B* deficiency affects *BRCA1, ATM* and *ATR* protein stability, which may lead to the defect of DNA repair machinery and, ultimately, uncontrolled cancer growth [21].

We have previously demonstrated that *INPP4B* and *RAD50* collectively affect breast cancer survival at the transcriptional and translational levels [22]. To further identify the synergies between *INPP4B* and *RAD50* on clinical consequences at the genetic level, we are motivated to identify the relevant disease-associating SNPs that affect the expression of each gene and are collectively prognostic of the clinical outcome of breast cancer patients.

## Materials and methods

**Figure 1:**
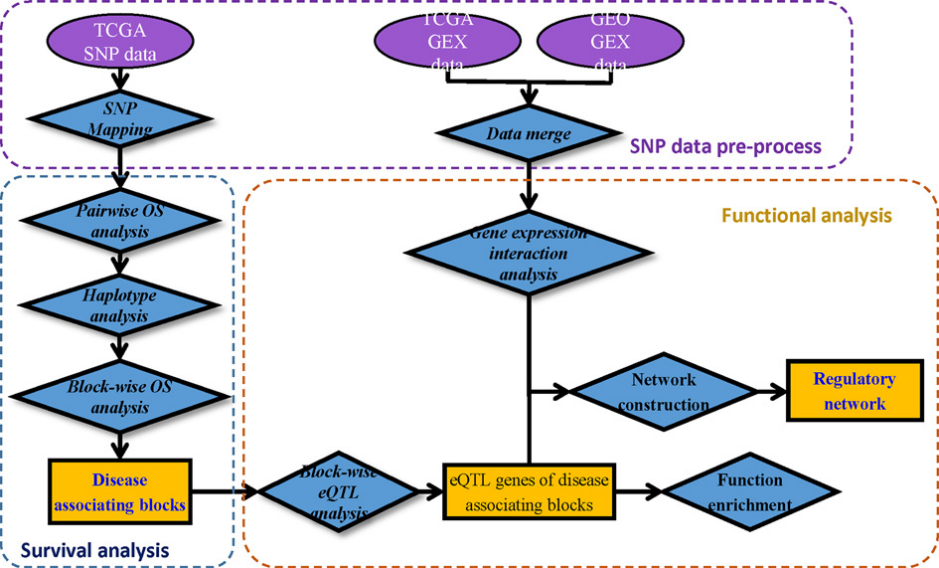
Schematic diagram of the analytic flow in the present study The purple oval and blue rhombi represent the SNPs in TCGA and analytical methods, respectively. Rectangle represents obtained results at each stage of analysis, where green represents ‘genes’, yellow represents ‘SNPs’, bronze represents ‘block’. Abbreviation: TCGA, The Cancer Genome Atlas.

The workflow of the methods is presented in Figure 1.

### Datasets

We retrieved 184485 SNPs of *INPP4B* and 19974 SNPs of *RAD50* from the dbSNP NCBI database [23]. Among these SNPs, 269 SNPs of *INPP4B* and 15 SNPs of *RAD50* were mapped to the Affymetrix SNP6.0 Array which was used in The Cancer Genome Atlas (TCGA). Information of the 284 SNPs covering 501 samples was retrieved from TCGA (http://cancergenome.nih.gov) and used for the downstream analysis. The gene expression data were retrieved from TCGA bioportal (http://www.cbioportal.org/), which contained 20440 genes and 1102 samples.

The GSE24450 dataset containing gene expression and clinical information of 183 breast tumors from the Helsinki University Central Hospital was retrieved from GEO with 10-year follow up information included in this dataset [24,25].

TCGA cohort and GEO cohort were merged followed by log2 transformation and batch correction using the ‘ComBat’ function in ‘sva’ package (version 3.30.1) in R [26]. These merged data were used in expression Quantitative Trait Loci (eQTL) analysis and pair-wise expression survival analysis.

### Pair-wise SNP survival analysis

We conducted breast cancer overall survival (OS) analysis on interactions between SNPs of *INPP4B* and *RAD50* using the Cox’s proportional hazard model. The recessive model was used in the pair-wise SNP association analysis, where the heterozygote was combined with the common homozygote assuming that the disease-associating phenotype is caused by the concomitant presence of both rare alleles in both interacting SNPs. The 10-year breast cancer OS survival analysis utilizing the ‘survival’ package (version 2.44.1.1) [27]. An SNP pair was considered interactive if the *P*-value of the Cox repression model was <0.05, the *P*-value of the interaction term was <0.05, and the number of iterations showing the model convergence rate was <10.

### Block-wise SNP survival analysis

Haplotype block refers to the inheritance of a cluster of SNPs [28]. LDlink (https://analysistools.nci.nih.gov/LDlink/) was used to calculate pair-wise linkage disequilibrium (LD) among SNPs associated with the same gene. SNPs with *r*^2^ greater than 0.8 were considered linked to the same haplotype. Non-LD linked SNPs were considered independent. We randomly selected one SNP among its LD-linked peers, and grouped independent SNPs of *INPP4B* and *RAD50* into distinct disease-associating blocks, respectively.

Block-wise survival analysis was performed between the disease-associating blocks of *INPP4B* and *RAD50*, respectively, assuming that the presence of the rare allele of any SNP within the block contributes equally to the synergistic clinical association. PredictSNP2 [29], a unified platform for predicting SNP effect, was employed to analyze the functionalities of non-LD linked SNPs. SNP2TFBS tool was used to check whether an SNP affects transcription factor binding site affinity [30].

### eQTL analysis

To identify genes whose expressions are significantly affected by the identified SNPs in each disease-associating block or the disease-associating block as a whole, we conducted the eQTL analysis. In single SNP eQTL analysis, gene expression was modeled against the allele status of an SNP using logistic regression. SNPs with a *P*-value of the linear model <0.01 were considered eQTLs of a gene. SNPs significantly affecting patient OS and expression of the gene it resides in were defined as disease-associating SNPs. In block-wise eQTL analysis, the allele statuses were combined and binarized such that concomitant presence of all rare alleles in a disease-associating block was considered as ‘1’ and block alleles containing any common allele was considered as ‘0’. Top genes filtered using *P*<0.01 and the coefficient β > 0.3 from the linear regression were selected as being significantly affected by the disease-associating block at the transcriptional level.

### Pair-wise expression survival analysis

We conducted breast cancer OS analysis on interactions between the expressions of a gene identified in the eQTL analysis to identify the quantitative association of a gene and its eQTLs using the Cox’s proportional hazard model. Gene expression of a gene was binarized by its median level, and the 10-year breast cancer OS survival analysis was conducted using the ‘survival’ package [27]. A gene pair was considered interactive if the *P*-values of the Cox repression model and the interaction term were both 0.05.

In addition, the expression of one gene was stratified by that of another to assess the influence of one gene on another or the interactions between two genes at the transcriptional level. ANOVA test was used to assess the statistical significance with *P*<0.05 being the threshold.

### Functional analysis

In order to investigate the functional consequences introduced by SNPs, pathway enrichment analysis was performed using genes affected by SNPs with statistical significance. Gene Ontology (GO) term, KEGG pathway and Reactome pathway were enriched using the web interface ‘Metascape’ [31]. Genes identified from the enriched pathways were collected for gene regulatory network construction using GeneMANIA (http://www.genemania.org) [32]. GeneMANIA uses the label propagation algorithm to predict gene–gene interactions at 7 levels (co-expression, co-localization, genetic interaction, physical interaction, shared protein domain, pathway and predicted) where interactions from genetic interaction are selected to construct our network. It outputs a regulatory network using the user-defined gene list based on databases and publications from multiple resources [32].

## Results and discussion

### SNPs of *INPP4B* and *RAD50* synergistically affect breast cancer survival

Multivariate Cox regression model was constructed to perform pair-wise interaction analysis using SNPs of *INPP4B* and *RAD50* on breast cancer OS. The results revealed nine SNPs, five from *INPP4B* (rs1219269, rs17016021, rs2636683, rs336298, rs9996933) and four from *RAD50* (rs3798134, rs3798135, rs2040704, rs2706347), having significant association with patient clinical outcome (Table 1).

**Table 1:** SNPs significantly affecting breast cancer OS

Gene	SNP	Position	Alleles	MAF	Consequence
*RAD50*	rs3798134	chr5:132629487	G>A	0.2564	Intron variant
	rs3798135	chr5:132629417	C>T	0.2556	Intron variant
	rs2040704	chr5:132637485	A>G	0.3297	Intron variant
	rs2706347	chr5:132569425	G>T	0.3095	Intron variant
*INPP4B*	rs1219269	chr4:142174118	A>T	0.2726	Intron variant
	rs17016021	chr4:142373233	A>T	0.0260	Intron variant
	rs2636683	chr4:142176930	C>T	0.3758	Intron variant
	rs336298	chr4:142094199	T>C	0.4002	Intron variant
	rs9996933	chr4:142087174	T>C	0.2676	Intron variant

‘MAF’ represents minor allele frequency.

### Concomitant presence of rare homozygotes of SNPs from disease-associating blocks is associated with poor breast cancer OS

**Figure 2:**
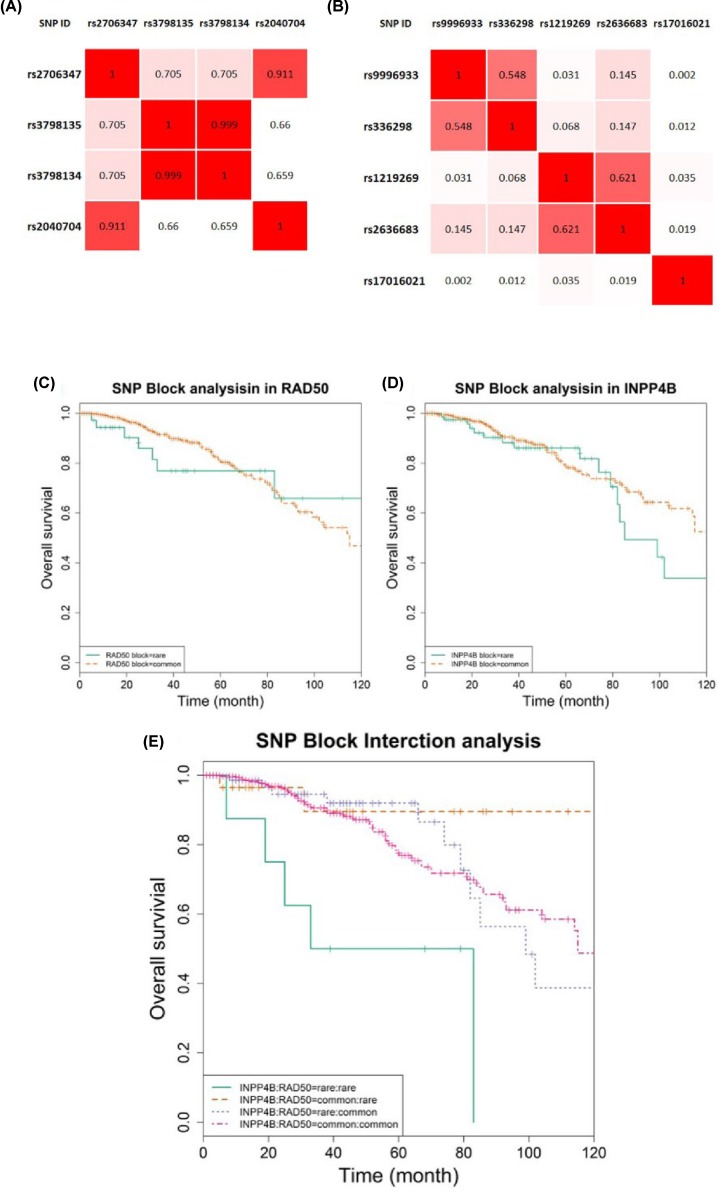
Interactions between SNPs in *INPP4B* and *RAD50* Heatmaps showing LD associations among SNPs of (**A**) *RAD50* and (**B**) *INPP4B*. OS analysis of (**C**) interactions of disease-associating block of *RAD50*, (**D**) disease-associating block of *INPP4B*, and (**E**) disease-associating blocks of *INPP4B* and *RAD50*. In subgraph (**E**), green curve represents concomitant presence of the rare homozygote of SNPs in both disease-associating blocks of *INPP4B* and *RAD50*; pink curve represents concomitant presence of the common homozygote of SNPs in both disease-associating blocks of *INPP4B* and *RAD50*; purple curve and bronze curve each represents the presence of the rare homozygote of SNPs in the disease-associating blocks of *INPP4B* and *RAD50*, respectively. The x-axis indicates the follow-up time, and the vertical axis shows the cumulative OS of breast cancer patients.

The four identified SNPs of *RAD50* are linked to two haplotypes, i.e. the *r*^2^ between rs2706347 and rs2040704 is 0.911, and that between rs3798135 and rs3798134 is 0.999 (Figure 2A). All SNPs of *INPP4B* are non-LD linked (Figure 2B).

Two disease-associating blocks were constructed where one SNP was randomly selected if multiple SNPs resided in one haplotype. That is, the *INPP4B* block includes rs336298, rs9996933, rs1219269, rs2636683, rs17016021, and the *RAD50* block contains rs3798134 and rs2040704. The results of the block-wise OS analysis using recessive model indicated that concomitant presence of the rare homozygote of any SNPs from each disease-associating block is associated with significantly reduced breast cancer OS (Figure 2E). The presence of all common homozygotes in either *INPP4B* or *RAD50* is sufficient to rescue patient clinical outcome.

On the other hand, either of the two disease-associating blocks drives significant differences on patient OS (Figure 2C,D), suggesting that it is the interaction between the two disease-associating blocks that differentiate breast cancer clinical outcome but not either one of the two blocks.

**Figure 3:**
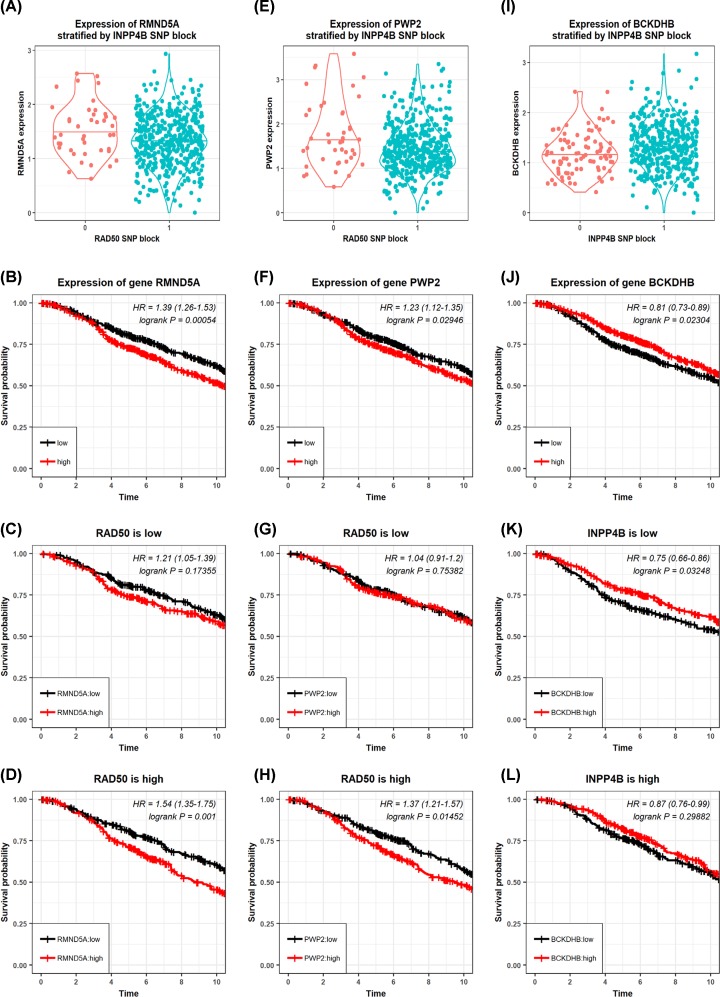
Associations between each disease-associating block and the corresponding gene (**A**) Correlation between *RMND5A* gene expression and allele status of the *RAD50* disease-associating block. The prognostic value of *RMND5A* on breast cancer OS (**B**) alone, (**C**) under low *RAD50* gene expression, (**D**) under high *RAD50* gene expression. (**E**) Correlation between *PWP2* gene expression and allele status of the *RAD50* disease-associating block. The prognostic value of *PWP2* on breast cancer OS (**F**) alone, (**G**) under low *RAD50* gene expression, (**H**) under high *RAD50* gene expression. (**I**) Correlation between *BCKDHB* gene expression and allele status of the *INPP4B* disease-associating block. The prognostic value of *BCKDHB* on breast cancer OS (**J**) alone, (**K**) under low *INPP4B* gene expression, (**L**) under high *INPP4B* gene expression.

**Figure 4:**
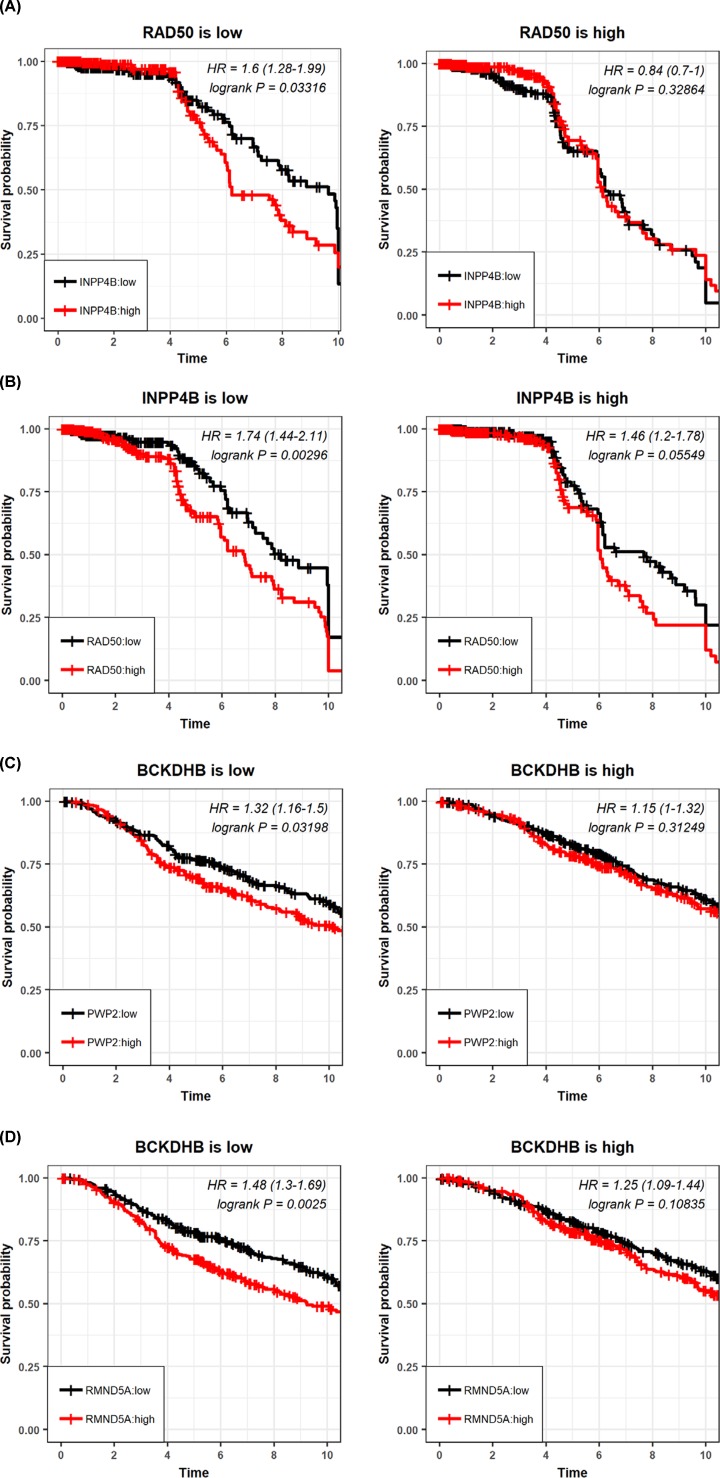
Pair-wise gene interactions (**A**) The prognostic value of *INPP4B* gene expression on breast cancer OS under high and low *RAD50* expression. (**B**) The prognostic value of *RAD50* gene expression on breast cancer OS under high and low *INPP4B* expression. (**C**) The prognostic value of *PWP2* gene expression on breast cancer OS under high and low *BCKDHB* expression. (**D**) The prognostic value of *RMND5A* gene expression on breast cancer OS under high and low *BCKDHB* expression.

We performed the eQTL analysis followed by the pair-wise expression survival analysis, which revealed that concomitant presence of the rare allele in the disease-associating block of *INPP4B* was positively associated with low *BCKDHB* expression that is risky (*P*=0.023, HR = 0.81, Figure 3), and that of *RAD50* was positively associated with *RMND5A* and *PWP2* high expression which were risky (*RMND5A*: *P*=0.0005, HR = 1.39; *PWP2*: *P*=0.029, HR = 1.23, Figure 3). In addition, *RMND5A* or *PWP2* overexpression was associated with breast cancer clinical outcome under *BCKDHB* low-expression, which were both risky (*RNMD5A* and *BCKDHB*: *P*=0.0025, HR = 1.48; *PWP2* and *BCKDHB*: *P*=0.03, HR = 1.32, Figure 4), and such a prognostic value diminishes under *BCKDHB* high expression. Therefore, the joint prognostic value of the two disease-associating blocks was in agreement with those from the pair-wise joint expression between *BCKDHB* and *RMND5A, BCKDHB* and *PWP2.* Further, *BCKDHB* interacts with *INPP4B*, where low *BCKDHB* under low *INPP4B* expression was risky (*P*=0.03, HR = 0.75, Figure 3), high *RMND5A* or *PWP2* was risky under high *RAD50* expression (*RMND5A*: *P*=0.001, HR = 1.54; *PWP2*: 0.015, HR = 1.37, Figure 3). On the other hand, low *INPP4B* and high *RAD50* expression are risky (*P*=0.00296, HR = 3.15, Figure 4), and high INPP4B and low RAD50 convey unfavorable clinical outcome (*P*=0.03, HR = 1.6, Figure 4).

### Gene function analysis of non-LD linked SNPs

**Figure 5:**
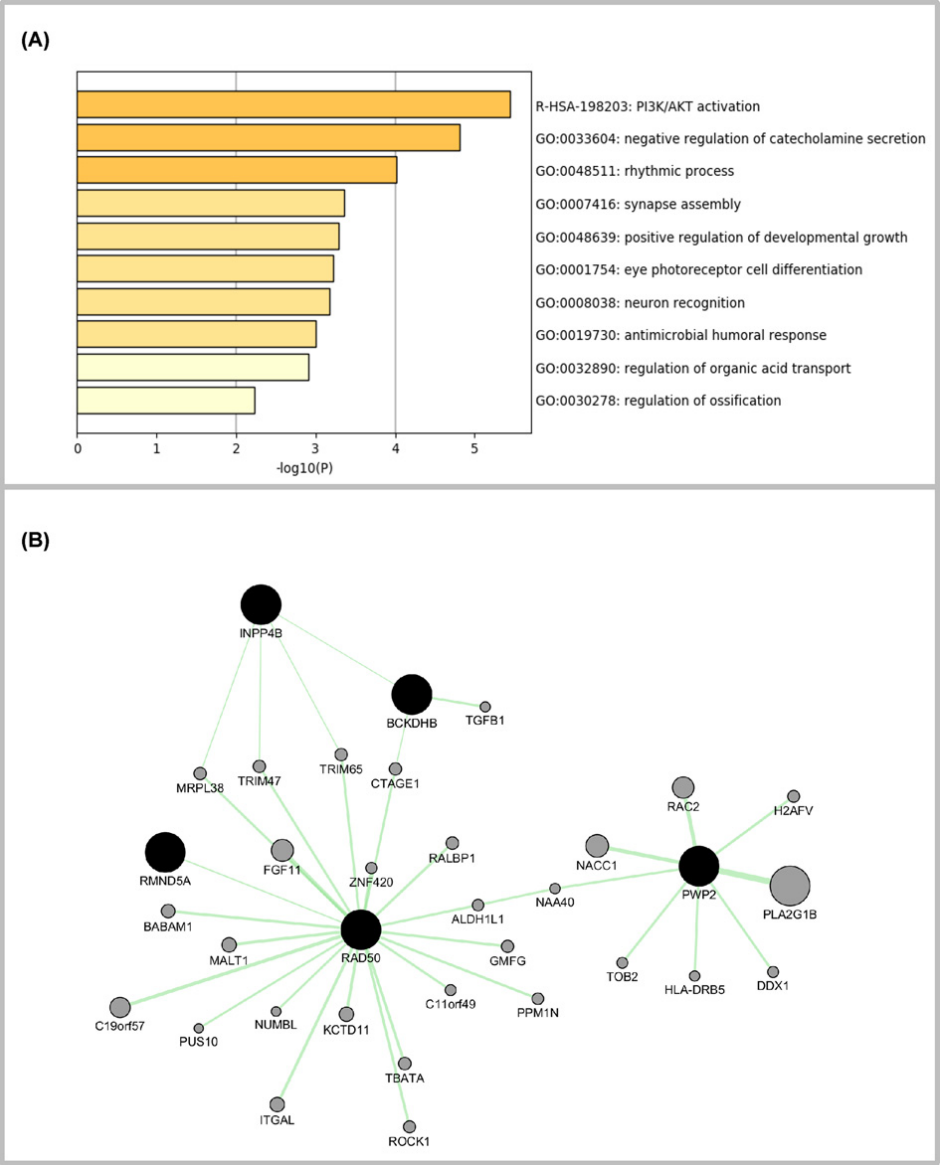
Pathway enrichment and network construction using genes quantitatively associated with *INPP4B* and *RAD50* disease-associating blocks (**A**) GO and KEGG enrichment analysis. (**B**) Network constructed using GeneMania where only genetic interactions were preserved.

We obtained 89 genes from the eQTL analysis, whose expression were significantly affected by the allele status of identified non-LD linked SNPs. GO and KEGG gene enrichment analysis showed that these genes were significantly enriched in ‘PI3K/AKT activation process’ (Figure 5A).

The regulatory network involving *INPP4B* and *RAD50* showed that *BCKDHB* and *INPP4B*, as well as *RMND5A* and *RAD50* have direct genetic interactions, PWP2 and RAD50 have indirect genetic interactions via *ALDH1L1* and *NAA40* (Figure 5B).

## Discussion

Through pair-wise and block-wise interactive OS analyses on SNPs of *INPP4B* and *RAD50*, we identified two disease-associating blocks, each containing five SNPs (*INPP4B*) and two SNPs (*RAD50*), respectively, which synergistically affect breast cancer clinical outcome. Specifically, concomitant presence of the rare homozygotes of all SNPs within each block is associated with decreased breast cancer OS, while neither one of these blocks differentiate breast cancer clinical outcome with statistical significance. Both *RAD50* and *INPP4B* are crucial to maintain genomic integrity and prevent tumorigenesis [7,12,13], and co-deletion of both genes commonly co-occurs in many types of cancers including breast cancer [10]. Our results further consolidate our understandings on cells and carcinogenesis, i.e. cells are robust systems having multiple ways to suppress tumorigenesis, and carcinogenesis is likely to occur when all tumor suppressive systems are dysfunctional.

The two disease-associating SNP blocks were each associated with the expression of genes that interacted with *INPP4B* and *RAD50*, respectively, with consistent directions regarding their prognostic values. For instance, concomitant presence of rare alleles in the disease-associating block of *INPP4B* was positively associated low *BCKDHB* expression, and the rare status of the *RAD50* disease-associating block was positively correlated with high *RMND5A* or *PWP2* expression. Importantly, concomitant presence of rare statuses of *INPP4B* and *RAD50* was risky, which was consistent with joint low *BCKDHB* and high *RMND5A* or *PWP2* expression (risky). This makes it possible to associate interactions at the genetic level with interactions at the gene expression level regarding clinical outcomes. Meanwhile, *BCKDHB* interacted with *INPP4B, RMND5A* and *PWP2* each interacted with *RAD50*, and *INPP4B* interacted with *RAD50* regarding clinical associations, and these clinical associations shared consistent directions. That is, low *BCKDHB* and low *INPP4B* is risky, high *RMND5A* and high *RAD50* is risky, low *BCKDHB* and high *RMND5A* is risky, low *INPP4B* and high *RAD50*, where the clinical association of each of these genes is transferable among these pair-wise joint clinical associations. Provided the strong correlation between the *INPP4B* disease-associating block and *BCKDHB*, and *RAD50* disease-associating block and *RMND5A*, it is highly likely that *BCKDHB* and *RMND5A* reflected and/or mediated interactions between *INPP4B* and *RAD50* at the gene expression level which also applies for pair *BCKDHB* and *PWP2*. Indeed, we found from GeneMania that *BCKDHB* had genetic interactions with *INPP4B* and *RAD50*, respectively, which were previously reported by [32], and *RMND5A* and *PWP2* had known genetic interactions with RAD50, which were in agreement with what we observed in the present study and supported our findings. Interestingly, *BCKDHB* was also genetically associated with TGFβ1 that plays critical roles in TGFβ signaling and responsible for cancer stemness; and *PWP2* genetically interacted with *RAD50* via *ALDH1L1* that is associated with neural stem cells *in vivo* [33], suggesting a trilateral connection among uncontrolled cell proliferation (as represented by INPP4B and PI3K/Pten pathway), DNA damage repair (as represented here by RAD50), and cancer stemess (as featured by TGFβ-mediated signaling and ALDH1L1).

Among the three genes mediating interactions between *INPP4B* and *RAD50*, only *RMND5A* has known evidence which is a tumor vasculature-associated gene with transmembrane or secreted protein products identified through expression profiling of ovarian cancer vascular cells [34]. *BCKDHB* encodes the E1 β subunit of branched-chain keto acid dehydrogenase and is a multienzyme complex associated with the inner membrane of mitochondria [35]. *PWP2* is involved in humoral immunity [36]. Their relevance with cancer initiation and progression is worthy to be investigated. *INPP4B* and *RAD50* were also genetically connected via *TRIM47* and *TRIM65*, and TRIM family proteins are known players in innate immunity, and have recognized roles in carcinogensis [37]. These together suggest the involvement of immune response, metabolism and angiogenesis in mediating synergies between *INPP4B* and *RAD50*.

We further identified disease-associating SNPs in both disease-associating blocks. That is, both rs2040740 residing in the intron of *RAD50* and rs17016021 from the intron of *INPP4B* are deleterious; and the rare allele of rs2040740 is significantly associated with reduced *RAD50* expression. The minor allele frequency (MAF) of the *RAD50* disease-associating SNP (rs2040704) is the highest among all SNPs in the *RAD50* disease-associating block, which is ∼0.33 (Table 1), suggesting its prevalence. Actually all SNPs in the *RAD50* disease-associating block have a relatively high prevalence (i.e. ranging from 0.25 to 0.33, Table 1), indicating that the pathway represented by *RAD50* is more likely to be dysfunctional. However, the MAF of the *INPP4B* disease-associating SNP (rs17016021) is rather rare, i.e. 0.026 (Table 1), suggesting that dysfunction of the pathway represented by *INPP4B* is less likely to occur whose mutation functions as a pivotal switch toward enhanced cancer cell proliferation potential.

Importantly, PCR assays testing the polymorphisms of both SNPs in clinical materials that are associated with differential clinical outcomes are necessary before translating our discovery into clinics, and this would be our next endeavor to make.

As aforementioned, we hypothesize that synergies created from the identified disease-associating blocks of *INPP4B* and *RAD50* are related to cell progression and mutation accumulation that ultimately affect patient clinical outcome from our eQTL and pathway enrichment analyses. Such synergies may also involve altered immune response and cancer stemness. However, the exact underlying mechanism still awaits to be explored and experimentally validated.

## Conclusion

We identified two disease-associating blocks of *INPP4B* and *RAD50*, each containing five and two SNPs, respectively. Concomitant presence of any rare homozygote from each of the two disease-associating blocks is associated with decreased breast cancer survival, through disenabling breast cancer cell proliferation and DNA repair signaling pathways as represented by *INPP4B* and *RAD50*. Our study provides genetic evidence on the prognostic synergies between *INPP4B* and *RAD50* on breast cancer outcome and deepens our understandings toward cancer progression that ultimately facilitates cancer precision medicine.

## Abbreviations

eQTL: expression Quantitative Trait Loci
GEO: Gene Expression Omnibus
GO: Gene Ontology
HR: Hazard Ratio
KEGG: Kyoto Encyclopedia of Genes and Genomes
LD: linkage disequilibrium
MAF: minor allele frequency
OS: overall survival
SNP: single nucleotide polymorphism
TCGA: The Cancer Genome Atlas
